# First rib fracture and Horner’s syndrome due to a motor vehicle collision: a case report

**DOI:** 10.1186/2045-709X-21-22

**Published:** 2013-07-05

**Authors:** James Demetrious

**Affiliations:** 1 Private Practice, Demetrious Chiropractic Orthopedics, 4837 Carolina Beach Road, Suite 205, Wilmington, NC 28412, USA

**Keywords:** Horner’s syndrome, First rib, Fracture, Collision, Chiropractic, Dissection

## Abstract

A case of a first rib fracture and Horner's syndrome due to a motor vehicle collision is reported. Initial evaluation in a hospital emergency department and follow-up by a medical primary care physician failed to provide identification of the Horner’s syndrome. Careful assessment and review of the patient’s symptoms, signs and images revealed this uncommon and important neurologic case presentation. A brief discussion related to traumatic first rib fracture, Horner’s syndrome and arterial dissections of the neck is provided.

## Background

Making the diagnosis of Horner’s syndrome requires careful consideration due to congenital, acquired, traumatic, compressive, invasive and iatrogenic etiologies [1-7]. The differential diagnosis of Horner’s syndrome can be challenging due to possible disruption of sympathetic fibers of first, second or third order neurons [4,5]. Such lesions are variable in their presentation, scope and affect due to complex neuro-anatomic pathways and potential damage subject to the proximity of other anatomic structures.

The incidence of fracture of the first rib is uncommon and varied in its causation [8-10]. First rib fracture is rare possibly due to its protected anatomic location [11]. Such injuries are clinically important due to the proximity and potential compression of neighboring vascular and neurologic structures. In motor vehicle collisions, blunt trauma caused by sudden movement of the head that is abruptly stopped by a seatbelt or windshield may cause stress through scalene and sternocleidomastoid muscles producing bending strain through the first rib and resultant fracture [11].

This paper describes a case of a 72-year-old gentleman who experienced a first rib fracture and Horner’s syndrome due to a motor vehicle collision.

## Case presentation

A 72-year-old man presented to our chiropractic office eight-weeks following a vehicular accident. While driving, he suffered a collision with an oncoming vehicle in which his car was impacted on its front left quarter panel. He wore a seatbelt and did not lose consciousness due to the impact.

The patient was transported via ambulance to a local hospital where evaluation and extensive imaging was immediately performed and read by different attending radiologists. The attending emergency medical physician diagnosed a rib fracture and the patient was subsequently released from the hospital. The patient sought care with his medical primary care physician and received a prescription for pain medication for the first rib fracture and neck pain. No other recommendations or care were provided to the patient.

The patient’s wife reported that while visiting the patient at the hospital immediately following the car accident, she noted asymmetry and partial drooping of his right eyelid. No discussion of the drooping eyelid was made by the patient or his wife with the previous attending physicians.

The patient sought care in our chiropractic office eight weeks following the vehicular collision for assessment and care of persistent neck discomfort. His past history was negative for contributory medical, neurologic or ophthalmologic disorders. The patient’s vital signs were normal. He was alert and oriented.

Initial visual inspection revealed miosis and partial ptosis of the right eye. His right eye was not responsive to direct or consensual light. Cardinal fields of gaze were normal. The patient denied alteration of facial sensation or hemi-facial anhidrosis of the affected side. The remainder of the patient’s cranial nerve examination was normal. Neurologic assessment of the extremities revealed normal sensory, deep tendon reflexes and motor function. Pathologic reflexes were not elicited. Cerebellar, coordination and balance evaluations were negative. Otoscopic examination was normal.

Auscultation of the carotid and subclavian arteries revealed no bruits. The lungs were clear to auscultation. Globally decreased cervical range of motion and localized tenderness was noted at C7/T1. Palpation revealed tenderness of the first rib at the apex of the right lung. The patient reported localized discomfort at C7/T1 upon cervical compression, Spurling’s test and Valsalva maneuver. No radiating pain was elicited. No other abnormalities were identified during physical examination.

## Imaging

The following comprehensive imaging was performed upon initial evaluation at the hospital and read by different attending radiologists. All imaging reports and images performed following the vehicular collision were obtained from the hospital. A review of the radiology reports and careful over-read of all images was conducted in our office by the author:

**Figure 1:**
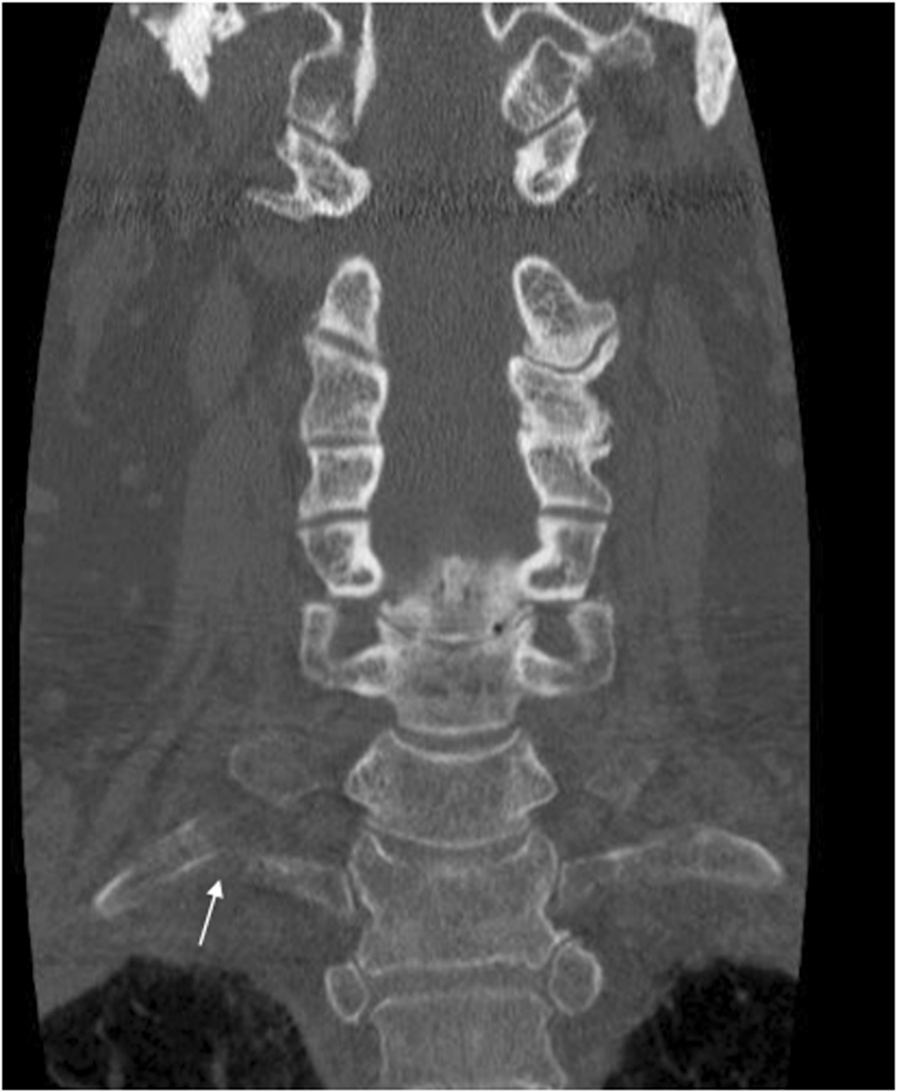
**Non-contrast CT of the cervical spine.** This coronal view reveals a displaced fracture of the proximal right first rib (white arrow).

**Figure 2:**
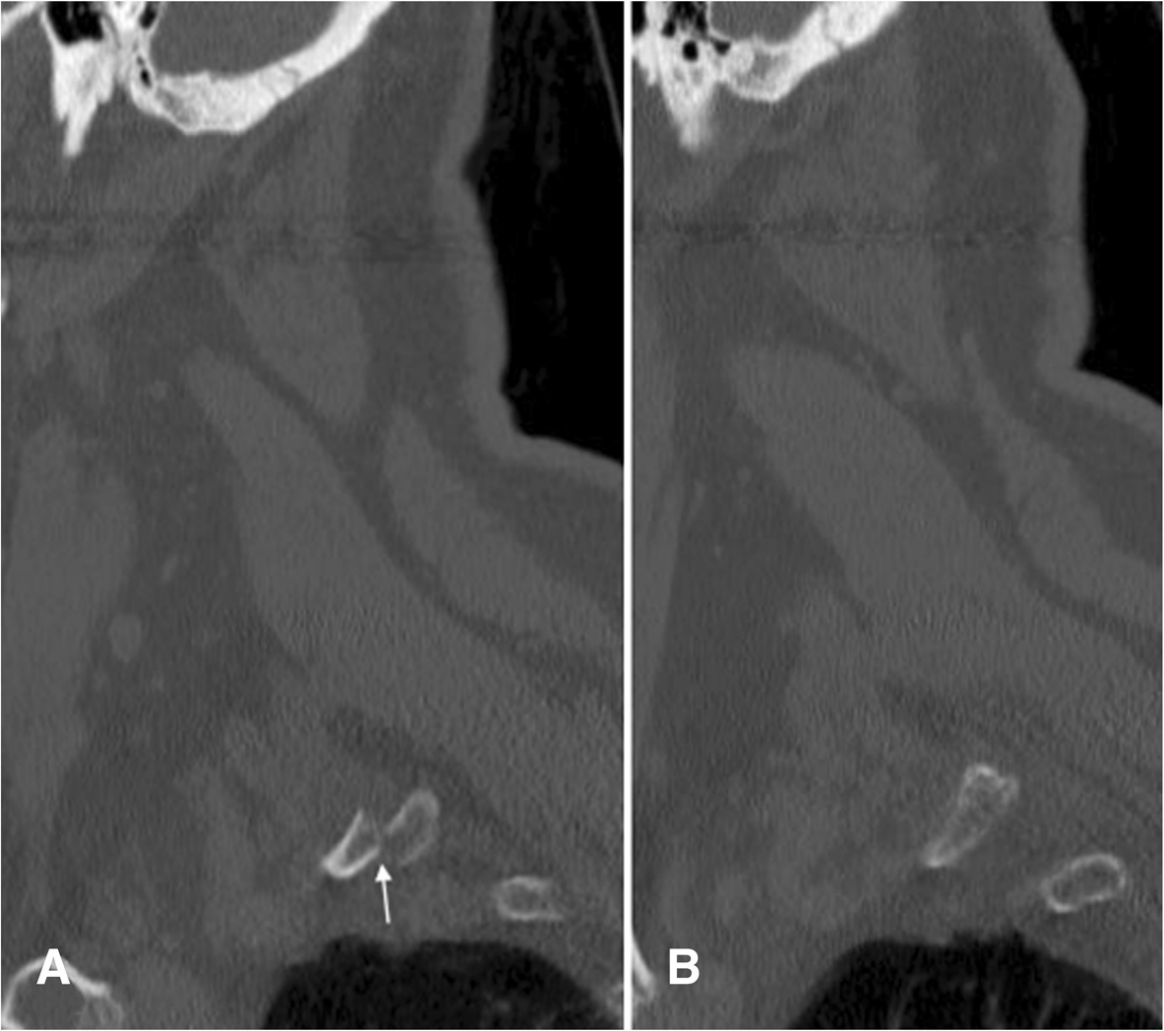
**Non-contrast CT of the cervical spine.** Sagittal image **(A)** reveals an anteriorly displaced fracture of the right first rib (white arrow). For comparison, image **(B)** reveals a normal left first rib.

**Figure 3:**
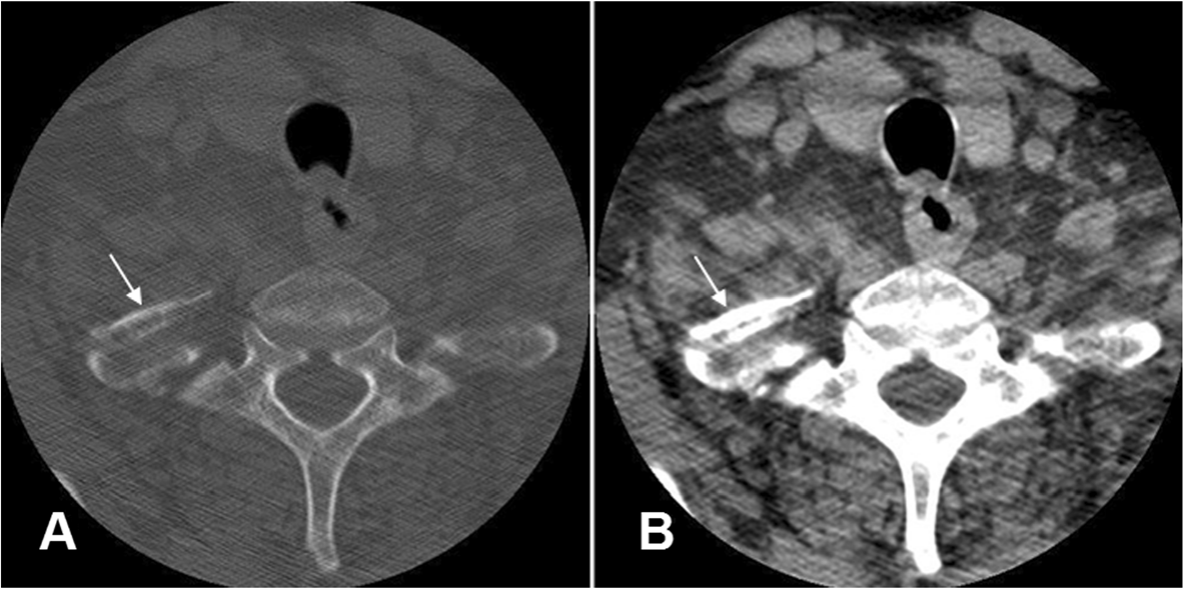
**CT of the thoracic spine with contrast.** The CT bone window image **(A)** and CT soft tissue window **(B)** reveal an anteriorly displaced rib fracture of the proximal right first rib (white arrows).

**Figure 4:**
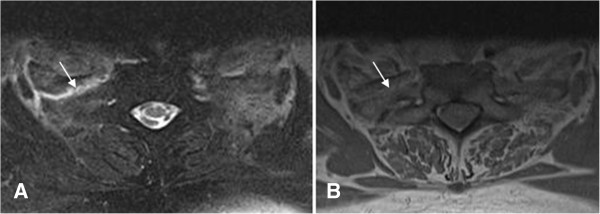
**Cervical spine magnetic resonance imaging MRI.** A T2 weighted image **(A)** reveals a displaced fracture at the proximal right first rib with increased signal intensity at the fracture site (white arrow). Image **(B)** reveals the displaced fracture at the proximal right first rib with isointense signal at the fracture site (white arrow). These findings are indicative of edema and hemorrhage.

**Figure 5:**
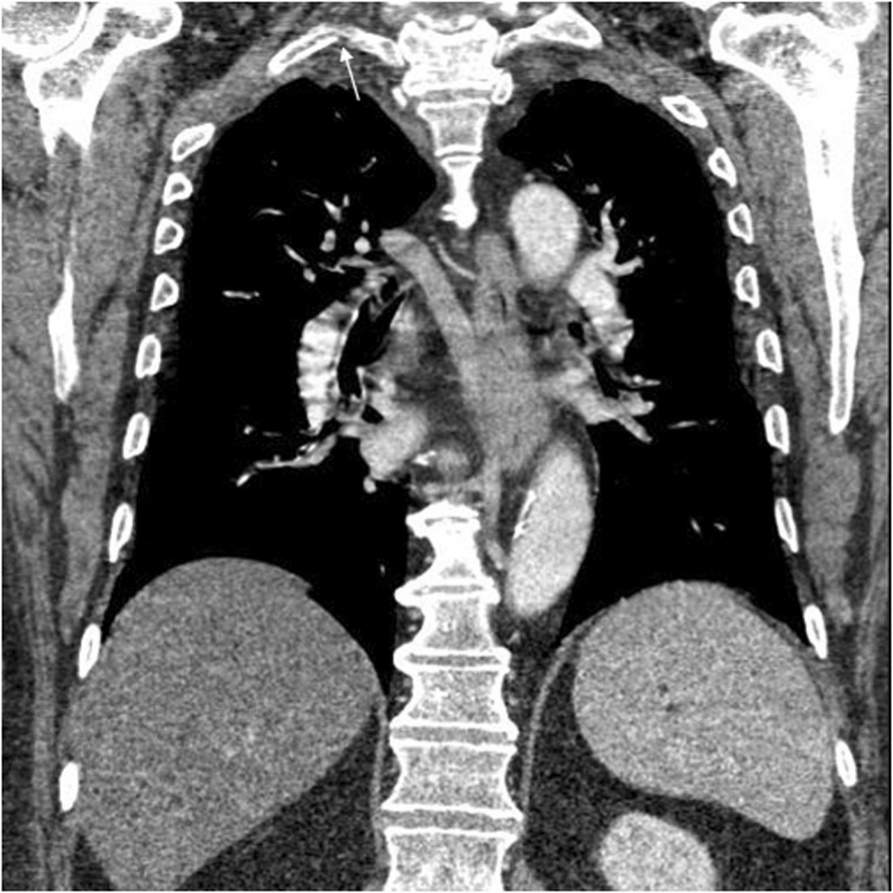
**CT of the chest with contrast.** This coronal view reveals a displaced fracture fragment of the proximal right first rib (white arrow).

**Figure 6:**
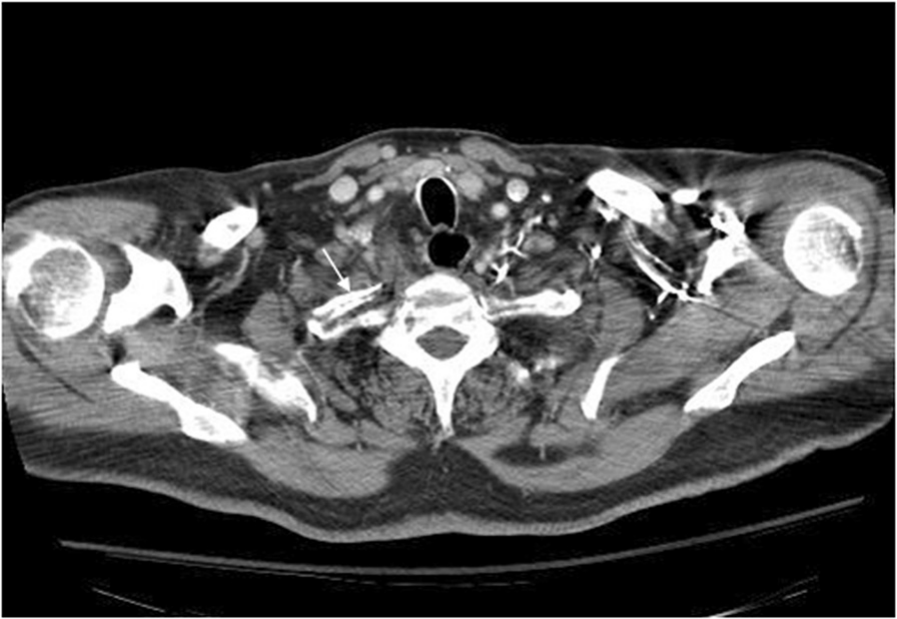
**CT of the chest with contrast.** This axial view reveals an anteriorly displaced fracture fragment of the proximal right first rib (white arrow).

• Computed tomography (CT) of the head without contrast revealed no acute findings.

• Non-contrast CT of the cervical spine with reformations revealed an acute, anteriorly displaced fracture of the proximal right first rib on coronal (Figure 1) and sagittal images (Figure 2).

• CT of the thoracic spine with contrast revealed an anteriorly displaced rib fracture of the proximal right first rib (Figure 3).

• An attending radiologist reported no acute abnormalities of the cervical spine magnetic resonance imaging (MRI) study. Subsequent over-read in our office by the author revealed a displaced fracture at the proximal right first rib with increased signal intensity at the fracture site on T2 weighted images (Figure 4).

• CT of the chest with contrast was performed. The attending radiologist failed to report the first rib fracture. Subsequent over-read in our office by the author revealed an anteriorly displaced fracture fragment at the proximal right first rib on coronal (Figure 5) and axial images (Figure 6).

Horner’s syndrome is present in up to 58% of internal carotid artery dissections [12]. Most patients experience neck, facial and head pain ipsilateral to the lesion because of ischemia or stretching of the trigeminal pain fibers surrounding the carotid arteries [13]. Ophthalmic manifestations have been reported to occur in up to 62% of patients with internal carotid artery dissection [13].

Due to the trauma sustained by the patient, persistent neck pain, the identified first rib fracture, persistent miosis and eyelid ptosis and concern for a traumatic arterial dissection, the patient was referred for a brain MRI and magnetic resonance angiography (MRA) examinations of the cervical and brain vessels [5,12,13]. He was also referred to a local neuro-ophthalmologist for assessment and follow-up.

The MRI and MRA studies were negative and the patient was provided a diagnosis of Horner’s syndrome due to first rib fracture. No specific treatment was rendered. Over the period of one year, the patient recovered fully.

## Discussion

The patient suffered Horner’s syndrome due to a traumatic, second order neuronal lesion. Disruption and edema at the fracture site likely produced an oculo-sympathetic palsy due to compression of the neighboring stellate ganglion and sympathetic fibers [14].

All of the advanced imaging studies performed visualized the fractured first rib. However, attending radiologists failed to note the fracture in two imaging reports. It is important to recognize the fallibility and inherent difficulty of identifying rib fractures via imaging modalities. Cho et al. report that missed rib fractures are not uncommon [15].

Despite the obvious visual cue of a drooping eyelid, the patient’s attending medical providers did not properly assess this condition. This is critical as the patient could have suffered a traumatic arterial dissection that may have been undetected and untreated for that time period [16,17]. Such an error could have been catastrophic.

Had the patient suffered an undiagnosed post-traumatic arterial dissection, subsequent delivery of chiropractic adjustment, spinal manipulation or manual therapy could have been wrongly implicated as a causative etiology of the dissection and/or Horner’s syndrome.

## Conclusions

In this uncommon case, a first rib fracture caused by the trauma of a motor vehicle collision produced Horner’s syndrome. While a ptotic eyelid might seem evident, it is possible as demonstrated in this case, to miss this important diagnosis. Horner’s syndrome can be difficult to assess and bears due attentiveness and diagnostic consideration.

## Consent

Written informed consent was obtained from the patient for publication of this case report and any accompanying images. A copy of the written consent is available for review by the Editor-in-Chief of this journal.

## Competing interests

The author declares no competing interests.

## Author’s information

The author is a Fellow of the Academy of Chiropractic Orthopedists. He conducts a private practice in Wilmington, NC, USA and teaches post-graduate chiropractic coursework pertaining to advanced differential diagnosis.
